# rs2357322 and rs17726078 Contribute to Breast Cancer Risk by Regulating *DLX2* Expression

**DOI:** 10.1155/tbj/3096873

**Published:** 2026-08-14

**Authors:** Xin-Xin Zhang, Hao Guo, Hai-Yan Li, Xi-Ting Zhou, Hong-Li Song, Jiang-Wei Xu, Yu-Hang Fu, Ru-Hui Tian, Ru Jiang, Chun-Chun Liu, Chang Sun

**Affiliations:** ^1^ College of Life Sciences, Shaanxi Normal University, Xi’an Shaanxi, 710119, China, snnu.edu.cn

**Keywords:** breast cancer, *DLX2*, expression regulation, rs17726078, rs2357322

## Abstract

**Background and Aims:**

Breast cancer is the most abundant cancer type in female. Genome‐wide association study suggests that rs2016394, one SNP at the intron of DLX2 divergent transcript (*DLX2-DT*), is significantly associated with this disease. Through 1000 genomes project data analysis, it is observed that another three SNPs, rs743605, rs2357322, and rs17726078, show strong linkage disequilibrium with rs2016394. However, the functional SNP(s) and mechanism are still unknown.

**Methods:**

Functional genomics effort was performed for this locus.

**Results:**

Through luciferase assay, it is disclosed that rs743605 and rs2016394 are not with the ability to alter gene expression. In contrast, rs2357322 and rs17726078 alleles present significantly different luciferase expression, thus suggesting that these two SNPs are functional mutations. Chromosome conformation capture indicates that *DLX2* (distal‐less Homeobox 2) can interact with the *cis*‐regulatory element containing these two SNPs and should be the regulatory target. Chromatin immunoprecipitation suggests that transcription factor MYC (MYC proto‐oncogene, bHLH transcription factor) can bind the rs17726078 surrounding region. Knock‐out of the segment containing these two SNPs by CRISPR/Cas9 can significantly increase *DLX2* mRNA and protein expression, cell proliferation, colony formation, migration, and wound healing, thus suggesting that the *cis*‐regulatory element is an attenuator for gene expression.

**Conclusion:**

rs2357322 and rs17726078 might influence *DLX2* expression and further contribute to breast cancer risk.

## 1. Introduction

Breast cancer is the most universally diagnosed malignant tumor in female, with ∼2.3 million new cases and ∼1.8 million deaths per year [1]. Breast cancer is influenced by multiple external and internal factors [2], including steroid hormone level, menarche and menopause age, reproductive history, diet, and adiposity. Among these, Genetic one plays an important role in occurrence of this disease. Indeed, a lot of genes have been observed to be with the ability to influence breast cancer onset, development, metastasis, and/or drug resistance [3, 4]. Besides these, epigenetic aberrations, which might be triggered by external environment and/or internal cellular microenvironment, can also alter the breast cancer process [5].

To identify the potential genetical contribution to this disease, multiple genome‐wide association studies (GWAS) have been conducted in different populations, including Caucasian, South African [6], Chinese [7], and Tunisian [8]. Therefore, hundreds of susceptibility loci have been identified (GWAS catalog, https://www.ebi.ac.uk/gwas/), such as rs7181788 [6], rs12493607 [7], rs13329835 [8], and rs10069690 [9]. Since most genetic markers associated with breast cancer are located at the noncoding region in human genome [10], it looks that the different risk among individuals should result from the *cis*‐regulatory element(s) and the mutation(s) within them instead of nonsynonymous ones, which has been verified by Capture Hi‐C and bioinformatics analysis [11]. Therefore, disclosure of these *cis*‐regulatory element(s) can provide more insight into the onset of breast cancer [12].

Among the SNPs affecting breast cancer risk in GWAS, one SNP at 2q31.1, rs2016394 [13], is located at the intron of a long noncoding RNA (lncRNA), DLX2 divergent transcript (*DLX2-DT*), which hints that this lncRNA might be a novel susceptible gene for this disease. However, the function of *DLX2-DT* in breast cancer has never been investigated. Moreover, since 500 k microarray was used in GWAS to represent whole human genome, the real functional mutation(s) for breast cancer might be not rs2016394 but other SNP(s) in linkage disequilibrium (LD) with it [14]. However, the LD pattern at this locus and this issue have not been scrutinized.

In this study, the potential functional mutations and susceptible gene were identified by LD and functional genomics approach. Their role on breast cancer onset was evaluated by genome editing.

## 2. Materials and Methods

### 2.1. LD Analysis

The genotype data of CEU (Utah Residents with European Ancestry), CHB (Han Chinese in Beijing), and YRI (Yoruba in Ibadan) surrounding rs2016394 were obtained from 1000 Genomes Project (1KGP). The LD pattern was calculated by ldSelect (https://droog.gs.washington.edu/ldSelect.html).

### 2.2. Plasmid Construction

The surrounding regions of the four SNPs, rs743605, rs2357322, rs2016394, and rs17726078, were amplified by primers listed in Table S1. After *Kpn*I and *Nhe*I (NEB, Ipswich, MA) treatment, the PCR products were ligated with proper vectors (see Table S1; Promega, Madison, WI). The plasmids with another allele were generated by mutagenesis with MutanBEST Kit (Takara, Dalian, China) and primers in Table S2. All recombinant plasmids were confirmed by sequencing.

### 2.3. Tissue Culture and Dual‐Luciferase Assay

MCF‐7 and 293TN cell lines were maintained in Dulbecco’s Modified Eagle Medium (DMEM; high glucose) with 10% fetal bovine serum (FBS; Biological Industries, Cromwell, CT). At the starting of this work, the cell passage numbers were 8 and 6 for MCF‐7 and 293TN, respectively.

When cell confluency was 80%, the recombinant plasmids (475 ng) were transfected into MCF‐7 cells along with pRL‐TK (Promega; 25ng) by lipofectamine 2000 (Thermo Fisher, Waltham, MA). After 48 h, cells were lysed, and luciferase expression was detected by dual‐luciferase reporter assay system (Promega). Six independent transfections were performed for each plasmid.

### 2.4. Chromosome Conformation Capture (3C)

3C was conducted as described in previous literature [15]. In brief, ∼10^8^ MCF‐7 cells were cross‐linked and lysed. The bacterial artificial chromosome (BAC) RP11‐1125C19 was used as a control. The cell chromatin and BAC (20 μg) were digested by *EcoR*I (NEB) and ligated by T4 DNA ligase (NEB). The DNA ligation efficiency was evaluated by quantitative PCR (qPCR) with iQ SYBR green (Bio‐Rad, Hercules, CA) and primers listed in Table S3.

### 2.5. Reverse‐Transcript PCR (RT‐PCR)

Total RNA was purified by TRIzol (Thermo Fisher) from ∼5 × 10^6^ MCF‐7 cells. cDNA was reverse‐transcribed by the RevertAid First Strand cDNA synthesis kit (Thermo Fisher). *DLX2-DT* and glyceraldehyde‐3‐phosphate dehydrogenase (*GAPDH*) was amplified by primers in Table S4. The amplicons were separated by electrophoresis on 3% agarose gel.

### 2.6. RNA‐Seq Analysis

RNA‐seq data for lymphoblastoid cell line (LCL) [16] and breast tissues [17] were downloaded from SRA database. *DLX2* (Genbank ID NM_004405.4) and *DLX2-DT* (NR_126376.1) expression was determined as described [18]. The genotype for LCL was obtained from 1KGP, and linear regression analysis was conducted between genotype and *DLX2* expression by SPSS 20.0 Statistics (IBM, Armonk, NY).

### 2.7. Chromatin Immunoprecipitation (ChIP)

ChIP was performed as previously described [15] for the rs17726078 surrounding region. Briefly, ∼5 × 10^6^ MCF‐7 cells were cross‐linked, lysed, and sonicated. The transcription factor (TF)/chromatin complex was immunoprecipitated by anti‐MYC (MYC proto‐oncogene, bHLH TF) antibody (Santa Cruz Biotechnology, Dallas, TX; #sc‐40) and normal mouse IgG (Santa Cruz Biotechnology; # sc‐2025). DNA was quantified by qPCR using primer pair CGCCACCACCATCACCACAAC and TTCCCCAAGGCCACAAAGTCTG.

### 2.8. Electrophoretic Mobility Shift Assay (EMSA)

EMSA was conducted as described in previous literature [15] with the probes listed in Table S5. In brief, probes were labeled with biotin, annealed, and incubated with MCF‐7 nuclear proteins. The probe/protein complex was separated by electrophoresis on 5% polyacrylamide gel, transferred to nylon membrane (Beyotime, Shanghai, China), and stained by streptavidin‐horseradish peroxidase (Beyotime).

### 2.9. *Cis*‐Regulatory Element Knock‐Out by CRISPR/Cas9

Small guide RNA (sgRNA) is listed in Table S6 and inserted into lentiCRISPRv2. Lentivirus was packaged by transfection into 293TN cell and utilized to infect MCF‐7 cell. The single clone with knock‐out was obtained by dilution and validated by PCR and sequencing with primer pair CTCACCGCAGCCTCGCAACT and GCCCGAGAACGCCGAGTGTC. The empty vector was also packaged and transfected to produce a control cell strain.

### 2.10. *DLX2* mRNA and Protein Expression and Cell Phenotype Measurement

Total RNA and cDNA library were prepared as abovementioned. *DLX2* mRNA and protein expression was evaluated by qPCR with primers in Table S4 and western blot, respectively. In qPCR, *GAPDH* was utilized as an internal control, and *DLX2* expression was calculated by 2^−ΔΔCt^, in which ΔΔCt is the threshold cycle difference between *GAPDH* and *DLX2*. In western blot, primary antibodies for DLX2 and tubulin were purchased from Proteintech (Wuhan, China; #26244‐1‐AP) and Abways (Shanghai, China; #AB0039), respectively.

Cell phenotypes, including proliferation, colony formation, migration, and wound healing, were determined as previously described [15].

### 2.11. Statistical Analysis

The luciferase expression, 3C and ChIP enrichment, *DLX2* expression, and cell phenotypes were displayed as mean ± standard deviation and compared by independent two‐sided Student’s *t*‐test in SPSS 20.0. *p* < 0.05 was supposed to be statistically significant.

### 2.12. Ethics Statement

This work was granted an exemption from requiring ethics approval by the Ethics Committee of Shaanxi Normal University since no human subjects were involved.

## 3. Results

### 3.1. LD Pattern Surrounding rs2016394

Within the 200‐kb surrounding region of rs2016394, there are 1085, 1116, and 1426 genetic variants in CEU, CHB, and YRI populations, respectively. Among them, only two SNPs, rs743605 and rs17726078, show strong LD (*r* ^2^ > 0.8) with rs2016394 in all three populations (Figure S1). Besides these, another SNP, rs2357322, present complete LD in CHB (*r* ^2^ = 1) and moderate LD in CEU and YRI (*r* ^2^ = 0.586 and 0.668, respectively; Figure S1). Consequently, these four SNPs constitute two dissimilar core haplotypes (Table S7) and might be potential functional SNP(s) for breast cancer. Since G of rs2016394 is the risk allele in GWAS [13], the common haplotype should be associated with high breast cancer risk.

### 3.2. Function of the Four SNPs

**FIGURE 1:**
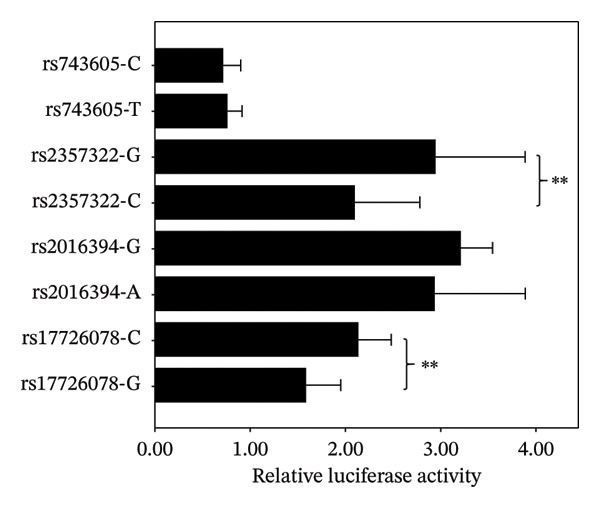
Luciferase expression for different alleles in core haplotypes. Each bar indicates one recombinant plasmid. The *x*‐axis denotes relative luciferase activity. For each SNP, the above and below alleles are from common and rare haplotype, respectively. All results are normalized by the result of pGL3‐promoter. ^∗∗^ *p* < 0.01.

To reveal the function of the four SNPs in breast cells, we ligated the surrounding regions with proper vectors and transfected them into MCF‐7 cell line. Since rs743605 is located in the 5′ untranslated region of *DLX2* (−36 relative to translation start), its surrounding region was inserted into pGL3‐basic. The plasmid containing *DLX2* promoter presents ∼43‐fold higher luciferase expression than pGL3‐basic (results not shown), thus indicating a strong promoter activity. However, no significant promoter activity difference is observed between two alleles of rs743605 (*p* = 0.44; Figure 1).

Since other three SNPs are not locating at the promoter region of any known genes and might be located at the enhancer region, their surrounding region was inserted into pGL3‐promoter vector. For the tag SNP rs2016394, no significant luciferase expression difference is obtained (*p* = 0.22; Figure 1), thus suggesting that rs2016394 is also not the functional SNP for breast cancer. In contrast, the alleles in common haplotype for another two SNPs, i.e., G of rs2357322 and C of rs17726078, present ∼40.3% and ∼34.7% higher luciferase activity (*p* = 0.005 and < 0.001, respectively) than the ones from rare haplotype, respectively (Figure 1), thus suggesting that they should be the real functional SNPs for breast cancer.

### 3.3. Regulation Target of the *Cis*‐Regulatory Element

**FIGURE 2:**
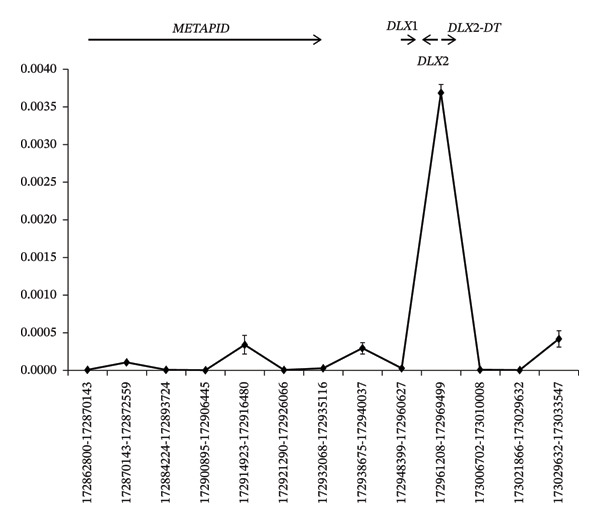
Ligation frequency between the *cis*‐regulatory element containing rs2357322 and rs17726078 and other genome regions in 2q31.1. The *x*‐axis designates the location of restrictive fragments in chr2, while the *y* axis shows the relative ligation efficiency. The above horizontal arrows indicate the illustrative location and transcript orientation of the genes in this region.

Since rs2357322 and rs17726078 are not locating at the promoter region of known genes, they might affect through a loop model. Therefore, we utilized 3C to search for the potential target gene(s). The constant primer was set to anchor the restrictive fragment containing rs2357322 and rs17726078. In contrast, the target primers were intended to interact with the restrictive fragments containing methionyl aminopeptidase Type 1D (*METAP1D*), distal‐less Homeobox 1 (*DLX1*), and *DLX2*/*DLX2-DT* promoter and 10 random selected genome segments. *DLX2* and *DLX2-DT* are in opposite transcript direction, and the distance between their transcript start is only 106 bp. That is, these two genes use the different strands of the same segment as their promoter, thus appearing at the same position in our 3C assay. As shown in Figure 2, ligation frequency is not increased in *METAP1D* (the 1st point, ∼109.1 kb away from the *cis*‐regulatory element) and *DLX1* (the 9th point, ∼23.7 kb away) promoter regions. In contrast, the *DLX2*/*DLX2-DT* promoter (the 10th point, ∼5.3 kb away) displays an evidently high interact efficiency (*p* < 0.001; one sample two‐sided *t*‐test), thus indicating that these two genes are close to the *cis*‐regulatory element in space and might be the regulating target.

### 3.4. Expression of *DLX2* and *DLX2-DT* in Breast Cell

In gene (https://www.ncbi.nlm.nih.gov/gene/) database, *DLX2-DT* is only expressed in testis, lung, intestine, stomach, and fetal brain (results not shown). To investigate its expression in breast cells, we performed RT‐PCR. Our primer pair for *DLX2-DT* was designed to anchor the same exon. As shown in Figure S2, this primer pair could amplify not cDNA but genomic DNA, thus indicating that *DLX2-DT* is not expressed in breast cells. We further downloaded and analyzed RNA‐seq data for breast cancer and paired adjacent nonmalignant tissues from 22 individuals [17], as previously described [18]. No *DLX2-DT* expression is observed in both normal and tumor tissues (results not shown). All these evidences indicate that *DLX2-DT* is absent in breast tissue, and the susceptible gene for breast cancer at this locus should be *DLX2*.

If *DLX2* is the susceptible gene for breast cancer, this gene should be differentially expressed between normal and breast tissues. We first compared *DLX2* expression in The Cancer Genome Atlas (TCGA) data by UALCAN (https://ualcan.path.uab.edu/index.html). The result indicates that *DLX2* is significantly overexpressed in breast invasive carcinoma (BRCA) than controls (*p* < 0.001; Figure S3).

We further investigated this issue in abovementioned paired breast cancer and normal tissues [17] and observed that *DLX2* expression increases ∼6.4‐fold on average (*p* = 0.03; paired two‐sided *t*‐test; Figure S4). All these facts suggest that *DLX2* should contribute to breast cancer onset and development.

### 3.5. Expression Quantitative Trait Loci (eQTL)

Given the function of rs2357322 and rs17726078 and their interaction with *DLX2*, these two SNPs should be eQTL for this gene. To substantiate this, we used the well‐established model, LCL [16], to investigate this issue. We utilized rs17726078 as a tag to represent the whole haplotype. As shown in Figure S5, *DLX2* mRNA level is significantly correlated with rs17726078 genotype (Pearson *r* = 0.20, *p* = 0.02; linear regression). Moreover, rs17726078 C allele is corresponding to a higher *DLX2* level, which is in agreement with our luciferase result. All these facts indicated that *DLX2* is the regulatory target of the *cis*‐regulatory element containing rs2357322 and rs17726078. *DLX2-DT* expression is also absent in LCL (results not shown), which cannot confirm or falsify the effect of the *cis*‐regulatory element on this lncRNA.

### 3.6. Interaction Between SNP and TF

**FIGURE 3:**
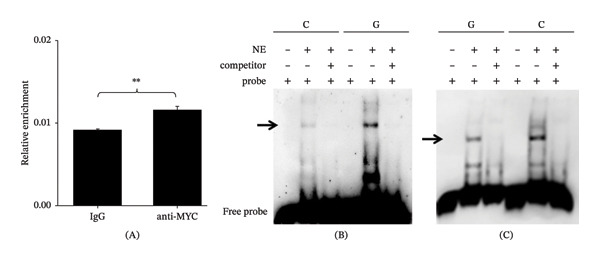
Interaction between rs2357322 and rs17726078 surrounding regions and TF. (A) The ChIP enrichment for rs17726078. ^∗∗^ *p* < 0.01. (B, C) EMSA result for rs2357322 and rs17726078, respectively. The two alleles are shown in above regions. NE represents nuclear extracts, while the arrow indicates protein/DNA complex.

Since rs2357322 and rs17726078 are located at the noncoding region, we hypothesized that they might be within the TF binding site and predicted the potential TF by Match at TRANSFAC (https://genexplain.com/transfac/). MYC was proposed to bind at the rs17726078 surrounding region. To validate this issue, ChIP was performed with anti‐MYC antibody. As shown in Figure 3A, the immunoprecipitated chromatin by anti‐MYC antibody is significantly enriched compared with IgG (*p* < 0.001), thus indicating that MYC can interact with the rs17726078 surrounding region. In contrast, the potential TF interacting with rs2357322 is still unknown.

To illustrate the binding affinity between rs2357322 and rs17726078 alleles, we further performed EMSA for both SNPs. As shown in Figure 3B,C, the two alleles of these two SNPs display evidently different binding affinity with nuclear extract. Moreover, the high expression alleles, i.e., G of rs2357322 and C of rs17726078, present higher binding affinity, which is consistent with our luciferase result.

### 3.7. Effect of the *Cis*‐Regulatory Element Knock‐Out

**FIGURE 4:**
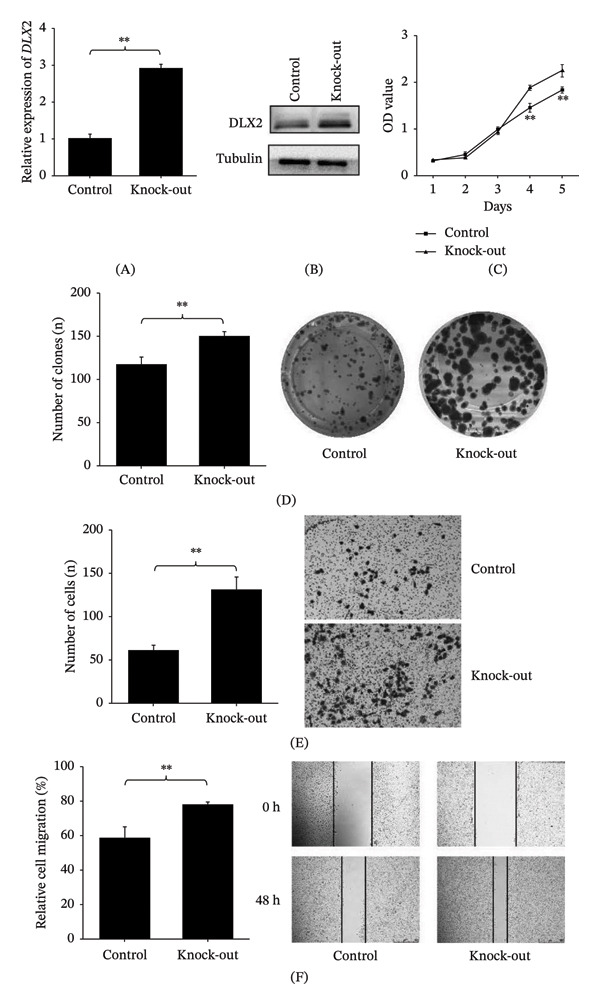
Effect of the *cis*‐regulatory element knock‐out on *DLX2* mRNA expression (A), protein expression (B), cell proliferation (C), colony formation (D), migration (E), and wound healing (F). For Part C, the *x*‐axis denotes culture time from seeding, while the *y*‐axis represents OD value. ^∗∗^ *p* < 0.01.

To further scrutinize the function of this *cis*‐regulatory element in gene expression and breast cancer onset, we knocked out the segment containing rs2357322 and rs17726078 by CRISPR/Cas9 (Figure S6) and measured *DLX2* expression and cell phenotype. As shown in Figure 4A, *DLX2* expression in knock‐out strain increases ∼1.90‐fold than control one (*p* = 0.001), thus indicating that the *cis*‐regulatory element is actually an attenuator. Consistent with this, the protein expression of DLX2 is also evidently enhanced in knock‐out strain (Figure 4B). Consequently, the cell proliferation (*p* < 0.001 for day 4 and 5), colony formation (*p* = 0.005), migration (*p* = 0.002), and wound healing (*p* = 0.008) are all increased in knock‐out strain (Figure 4C–F). All these facts suggest that the fragment containing rs2357322 and rs17726078 is a *cis*‐regulatory element for *DLX2* and further contributes to breast cancer onset and development.

## 4. Discussion

In the current study, the functional mutations and regulatory target were identified by population genetics and functional genomics approach. Their contribution to *DLX2* expression and breast cancer onset was validated by genome editing.

DLX2 is an important TF, and its regulation targets include multiple oncogenes, such as *MYC*, betacellulin (*BTC*) [19], snail family transcriptional Repressor 1 (*SNAI1*) [20], Wnt Family Member 1 (*WNT1*) [21], and Cadherin 2 (*CDH2*) [22]. Besides these, DLX2 can also reduce ATM (ATM serine/threonine kinase)‐p53 signaling [23]. All these facts indicated that *DLX2* is an important oncogene [24, 25], especially for triple‐negative breast cancer (TNBC) [26]. Considering all these facts, it is not surprising to observe that G of rs2357322 and C of rs17726078 can induce a higher *DLX2* expression than corresponding alleles and further contribute to breast cancer risk [13]. Moreover, it is necessary to point out that breast cancer can be divided into multiple subtypes, including Luminal A, Luminal B, human epidermal growth Factor 2 (HER‐2) positive, and TNBC. MCF‐7 is an estrogen receptor (ER) positive breast cancer cell line. Our expression analysis is based on ER‐positive breast cancer [17]. Therefore, there is no investigation on the role of *DLX2* and these two variants in HER‐2‐positive breast cancer and TNBC. Further data analysis on these subtypes or functional genomics work in related breast cancer cell lines that can shed more light on this issue.

The knock‐out by CRISPR/Cas9 indicates that the surrounding region of rs2357322 and rs17726078 can actually downregulate *DLX2* expression. The *cis*‐regulatory elements effecting as attenuator are not as common as enhancer but indeed existing in eukaryotic genome as shown in some studies [27, 28]. However, the interaction between this *cis*‐regulatory element and other *trans*‐ones is still unknown, which deserve further investigation. We also used ENCODE (https://www.encodeproject.org/) database to search for the potential histone modification. As shown in Figure S7, there are evidently H3K9me3 and H3K27me3, two common signals for transcriptional inhibition [29], at rs2357322 and rs17726078 surrounding regions in breast cancer cell lines HMEC and MCF‐7, which is consistent with the function of this *cis*‐regulatory element.

We proposed that rs2357322 and rs17726078 contribute to breast cancer risk. However, the distribution of these two SNPs in the case‐control group is still not known. To clarify this issue, we downloaded the original results from GWAS catalog. In study GCST004988 [13], the *p* values for rs2357322 and rs17726078 are 1.7 × 10^−8^ and 7.6 × 10^−12^, respectively, the latter of which is rather close to the tag SNP rs2016394 (6.2 × 10^−12^). Moreover, the search in Open Targets Genetics (https://genetics.opentargets.org) suggested that rs17726078 is significantly associated with breast cancer in multiple studies from FINNGEN and UK Biobank (see Table S8). All these facts support our proposal. Since case‐control cohort is not available to us, we cannot perform an independent verification for this issue. Further association analysis, especially for the multiple subtypes of breast cancer, might strengthen the effect of these two variants.

Our result suggests that it is not the tag SNP rs2016394 but rs2357322 and rs17726078 influencing breast cancer risk. Considering this fact, it looks that rs2357322 and rs17726078 might be better genetic markers to predict the probability of developing breast cancer than rs2016394. Indeed, the LD between rs2016394 and rs2357322/rs17726078 is high but not complete in representative populations.

Although rs2357322 and rs17726078 can influence breast cancer risk, they are only responsible for a small portion of breast cancer cases, just like other susceptible loci. Recent studies indicate that SNP–SNP interaction might contribute to breast cancer onset [30–32]. Indeed, multiple variants individually fail to show significant association or only present weak association with breast cancer. However, in specific combination, the haplotype is significantly associated with breast cancer predisposition [30–32], which emphasizes the importance of the crosstalk among variants. It is still unknown which genetic variant(s) can interact with rs2357322 and rs17726078 and further contribute to breast cancer onset, which deserves further investigation.

Besides rs2016394, there is another SNP in 2q31.1, rs1550623, and is also significantly associated with breast cancer in GWAS [13, 33, 34]. In CHB, rs1550623 is not polymorphic. In CEU and YRI, the *r* ^2^ between rs2016394 and rs1550623 is rather low, i.e., 0.029 and 0.006, respectively. Furthermore, although both genetic marks are within 2q31.1, the physical distance between them is as far as 1.24 Mb, which is beyond the range of our 3C assay. We further analyzed the association between rs1550623 genotype and *DLX2* expression in abovementioned dataset and no significant association is observed (Pearson *r* = 0.028, *p* = 0.28; Figure S8). All these facts suggest that rs1550623 is an independent GWAS signal and should be with different mechanism for the breast cancer association.

It has been proposed that *MYC* is the regulatory target of DLX2 [19]. Our effort suggested that MYC can also bind with the attenuator of *DLX2*. Therefore, these two genes might form a negative feedback in a regulatory network, which might play some roles in cell cycle and deserve further investigation.

There are multiple limitations in this study. First, the TF interacting with rs2357322 is still unknown, which leaves the mechanistic model partially unresolved. The bioinformatics analysis fails to disclose any known TFs, which hints that there might be an unknown TF surrounding rs2357322. Second, the function of rs2357322 and rs17726078 is investigated only in breast cancer cell line, and the eQTL analysis is performed only in LCL cell lines. There is no related analysis in breast cancer patients. Since the breast tissues are not available for us, we cannot provide these data. Some other technologies might better illuminate this in future.

Besides *DLX2* promoter, rs743605 is also located at the *DLX2-DT* promoter (−432 relative to transcript start), which indicates that rs743605 might alter *DLX2-DT* expression in some other tissues and further contribute to other phenotypes. Further analysis might provide more insight into this issue.

## 5. Conclusions

rs2357322 G and rs17726078 C alleles can increase *DLX2* expression and further contribute to breast cancer risk.

## Author Contributions

Xin‐Xin Zhang: investigation and writing–original draft. Hao Guo: investigation, formal analysis, and data curation. Hai‐Yan Li: investigation. Xi‐Ting Zhou: investigation. Hong‐Li Song: investigation. Jiang‐Wei Xu: investigation. Yu‐Hang Fu: investigation. Ru‐Hui Tian: investigation. Ru Jiang: investigation. Chun‐Chun Liu: investigation. Chang Sun: conceptualization, funding acquisition, writing–original draft, writing–review and editing, and project administration.

## Funding

This work was supported by the National Natural Science Foundation of China (No. 31370129) and Fundamental Research Funds for the Central Universities (GK202302003).

## Disclosure

All authors have read and approved the final version of the manuscript. Dr. Chang Sun had full access to all of the data in this study and takes complete responsibility for the integrity of the data and the accuracy of the data analysis. The funders had no role in the study design; data collection, analysis, and interpretation; writing of the report; and decision to submit the report for publication.

Dr. Chang Sun affirms that this manuscript is an honest, accurate, and transparent account of the study being reported; that no important aspects of the study have been omitted; and that any discrepancies from the study as planned have been explained.

## Conflicts of Interest

The authors declare no conflicts of interest.

## Supporting Information

Additional supporting information can be found online in the Supporting Information section.

## Supporting information


**Supporting Information** This supporting information provides additional information and data related to the study presented in the main manuscript. It includes detailed methodology and additional data analysis. This supporting information is essential for providing further evidence for the findings, supporting the conclusions, and presenting additional analyses. Figures and tables related to the supporting information are provided as separate files within the review system. These files are labeled as Table S1–S8 and Figures S1–S8 and contain detailed method and additional data analysis.

## Data Availability

The data that support the findings of this study are available from the corresponding author upon reasonable request.
