# Supplementary material for: rs2357322 and rs17726078 Contribute to Breast Cancer Risk by Regulating DLX2 Expression
Source: Breast J. 2026 Aug 14;2026:3096873. doi: 10.1155/tbj/3096873 (PMC13473913; doi:10.1155/tbj/3096873)
Supplement: Supplementary file 1 [file TBJ-2026-3096873-s001.docx]

Table S1. Primers in plasmid construction.

| SNP | Primer | Anneal temperature (ºC) | Vector |
| --- | --- | --- | --- |
| rs743605 | GGTACCGTCTCCACGCCTTCTGTCTTCCTG  GCTAGCCCTGGCCCGAGACGGGAAA | 60 | pGL3-basic |
| rs2357322 and rs2016394 | GGTACCCTCACCGCAGCCTCGCAACT  GCTAGCGGCAGCTTTTCACCATCTAAGGGG | 60 | pGL3-promoter |
| rs17726078 | GGTACCCCCAGGGGCTTGAACCGAAATC  GCTAGCGCCCGAGAACGCCGAGTGTC | 61 | pGL3-promoter |

Table S2. Primers for mutagenesis.

| SNP | Primer sequence^a^ | Anneal temperature (ºC) |
| --- | --- | --- |
| rs743605 | AGGTGGCGGGcGTGCGGGGGA  CTGCTCTTTCCCGTCTCGGGCC | 72 |
| rs2357322 | GGCTGTTTCCcGCCAGGGGAC  CAGAGCGACAGCGGCCTT | 68 |
| rs2016394 | CCTGGAAGGGgGGTTCCCCTG  ACTCTCTTTGGGGAGAGAGGG | 69 |
| rs17726078 | GATGTGCTGAgACGTGTGCTC  CTCTTCACATCCAAAGGAGG | 63 |

^a^Target site in lower case.

Table S3. Primers in 3C-qPCR.

| Name | Primer sequence | Location of Restrictive fragment^a^ | Location of primer | Functional elements |
| --- | --- | --- | --- | --- |
| C | TTTCCTCAAGTTTTCAGTAGCAGCTTCC | chr2:172972265-172983626 | chr2:172983548-172983575 | *cis*-regulatory element |
| T1 | GGGGTACAGGTAGTTTTTCGTTACATGG | chr2:172862800-172870143 | chr2:172870097-172870124 | *METAP1D* promoter |
| T2 | GGTTCATGGTCCTGCATTGCC | chr2:172870143-172872559 | chr2:172872475-172872495 |  |
| T3 | GGAAGGAGAGTGCTTGTTTTTAATGTCTG | chr2:172884224-172893724 | chr2:172893684-172893712 |  |
| T4 | GCTCATCATTGGTTAAATAAGGTGTTGTG | chr2:172900895-172906445 | chr2:172906399-172906427 |  |
| T5 | CAGGCTGCTGCCCTTCCTCTC | chr2:172914923-172916480 | chr2:172916390-172916410 |  |
| T6 | CGTTAGAGTAGAGTTTTCCATTGTCACGC | chr2:172921290-172926066 | chr2:172925983-172926011 |  |
| T7 | CTGTTAACGTTCGGCTGTCTGGAAC | chr2:172932068-172935116 | chr2:172935005-172935029 |  |
| T8 | GCTTGGGTTCATGGGCAGATG | chr2:172938675-172940037 | chr2:172939959-172939979 |  |
| T9 | CAGGATAGCGCCCCATCCTACAG | chr2:172948399-172960627 | chr2:172960576-172960598 | *DLX1* promoter |
| T10 | CTCGTAAGTAGCTGGGATTACAGGCG | chr2:172961208-172969499 | chr2:172969398-172969423 | *DLX2/DLX2-DT* promoter |
| T11 | CCCATCCTCCACCAACCCTATAAC | chr2:173006702-173010008 | chr2:173009943-173009966 |  |
| T12 | CAGAATAGAACCTGATGGGAGGTAATATGG | chr2:173021866-173029632 | chr2:173029555-173029584 |  |
| T13 | TGAGGATATGAGATTTAGAGGGGCCAG | chr2:173029632-173033547 | chr2:173033457-173033483 |  |

^a^Based on human genome build 37.

Table S4. Primers for RT-PCR or qPCR.

|  | Primer sequence | Amplicon length |
| --- | --- | --- |
| *DLX2* | GGAAACCCCGCACCATCTAC  TTCTTGAACTTGGACCGGCG | 169 |
| *DLX2-DT* | CTGCTTTGGATCGCTTGTCC  CTCGAGCCTCAGTGTCCATC | 133 |
| *GAPDH* | GAAGGTGAAGGTCGGAGTC  GAAGATGGTGATGGGATTTC | 226 |

Table S5. Probe in EMSA.

| SNP allele | Probe sequence^a^ |
| --- | --- |
| rs2357322 G allele | CTGGGCTGTTTCCGGCCAGGGGACGCG  CGCGTCCCCTGGCCGGAAACAGCCCAG |
| rs2357322 C allele | CTGGGCTGTTTCCCGCCAGGGGACGCG  CGCGTCCCCTGGCGGGAAACAGCCCAG |
| rs17726078 G allele | GAGGATGTGCTGAGACGTGTGCTCGGG  CCCGAGCACACGTCTCAGCACATCCTC |
| rs17726078 C allele | GAGGATGTGCTGACACGTGTGCTCGGG  CCCGAGCACACGTGTCAGCACATCCTC |

^a^Target site underlined.

Table S6. sgRNA sequence for CRISPR/Cas9.

| Position | Sequence | Genome location^a^ |
| --- | --- | --- |
| Left | CACCGTACAATATCACCCACATTG  AAACCAATGTGGGTGATATTGTAC | chr2:172972696-172972715 |
| Right | CACCGAGTGTCGCGGTAAACCTGG  AAACCCAGGTTTACCGCGACACTC | chr2:172974716-172974735 |

^a^Based on human genome build 37.

Table S7. SNPs in core haplotypes.

| rs ID | Position in chr2^a^ | Common haplotype | Rare haplotype |
| --- | --- | --- | --- |
| rs743605 | 172967302 | C | T |
| rs2357322 | 172972828 | G | C |
| rs2016394 | 172972971 | G | A |
| rs17726078 | 172974566 | C | G |

^a^Based on human genome build 37.

Table S8. Association between rs17726078 and breast cancer in GWAS.

| Study ID | Trait | *P* | Beta | Odds Ratio | Author |
| --- | --- | --- | --- | --- | --- |
| FINNGEN_R6_C3_BREAST | Malignant neoplasm of breast | <0.001 | -0.06 | 0.941765 | FINNGEN_R6 |
| NEALE2_20110_5 | Breast cancer \| illnesses of mother | <0.001 | -0.03271 | 0.967817 | UKB Neale v2 |
| SAIGE_174 | Breast cancer | 0.002 | -0.0414 | 0.959445 | UKB SAIGE |
| FINNGEN_R6_C3_BREAST_HER2NEG | Malignant neoplasm of breast | <0.001 | -0.05542 | 0.946088 | FINNGEN_R6 |

Fig S1. Pairwise LD pattern in CEU (A), CHB (B), and YRI (C) populations for rs2016394 surrounding region. Each square denotes a calculated *r^2^* between two SNPs in extension. The color, from white to black, represents *r^2^* value (from 0 to 1). The vertical arrow points out the position of rs2016394.

Fig S2. RT-PCR for *DLX2-DT*. The left lane is ladder (from top to bottom, 2000, 1000, 750, 500, 250, and 100 bp). The lane 1 is amplified from genomic DNA with *DLX2-DT* primer pair. The lane 2 is amplified from cDNA with *DLX2-DT* and *GAPDH* primer pairs. The above and bottom white arrows point out the position of *GAPDH* and *DLX2-DT* amplicon, respectively.

Fig S3. *DLX2* expression in BRCA and normal tissues in TCGA. The figure is from UALCAN (<https://ualcan.path.uab.edu/index.html>). The *x*-axis indicates two kinds of tissue and *y*-axis represent *DLX2* expression. Blue and red colors indicate normal and tumor tissues, respectively. Sample size is shown in bracket.

Fig S4. *DLX2* expression in paired normal and breast cancer tissues. The *x*-axis indicates two kinds of tissue and *y*-axis represent *DLX2* expression in FPKM (Fragments Per Kilobase of exon model per Million mapped fragments) unit. Sample size is shown in bracket.

Fig S5. Association between rs17726078 genotype and *DLX2* expression in LCL. The *x* axis designates rs17726078 genotype while *y* axis indicates *DLX2* expression (log transformed).

Fig S6. Schematic diagram for CRISPR/Cas9 assay design. Part A indicates the gene structure and transcript orientation of *DLX2* and *DLX2-DT*. Bar and dot line represent exon and intron, respectively. Part B points out the relative position of sgRNAs and SNPs. The green and red colors indicate gRNA and SNP, respectively. Part C is the sequencing result of knock-out cell strain. The sgRNA region is in blue background.

Fig S7. Histone modification surrounding rs2357322 (A) and rs17726078 (B) in breast cell line. The *x* axis indicates chr2 coordinate, while *y* axis shows the signal magnitude. The yellow vertical lines indicate the position of the SNPs. The pink color denotes the end of ChIP-seq peak.

Fig S8. Association between rs1550623 genotype and *DLX2* expression in LCL. The *x* axis designates rs1550623 genotype while *y* axis indicates *DLX2* expression (log transformed).
